# Augmented reality simulator for CT-guided interventions

**DOI:** 10.1007/s00330-021-08043-0

**Published:** 2021-06-10

**Authors:** D. Amiras, T. J. Hurkxkens, D. Figueroa, P. J Pratt, B. Pitrola, C. Watura, S. Rostampour, G. J. Shimshon, M. Hamady

**Affiliations:** 1 Imaging Department, Imperial College Healthcare NHS Trust, London, UK; 2 Department of Surgery, Imperial College London, London, UK; 3 Digital Learning Hub, Imperial College London, London, UK; 4 Imperial College London, London, UK; 5 Medical iSight Corporation, New York, USA; 6 Queen Mary University of London, London, UK

**Keywords:** Augmented reality, Simulation training, Image-guided biopsy, Tomography, X-ray computed

## Abstract

**Introduction:**

CT-guided interventions are taught using a mentored approach on real patients. It is well established that simulation is a valuable training tool in medicine. This project assessed the feasibility and acceptance of replicating a CT-guided intervention using a bespoke software application with an augmented reality head-mounted display (ARHMD).

**Methods:**

A virtual patient was generated using a CT dataset obtained from The Cancer Imaging Archive. A surface mesh of a virtual patient was projected into the field-of-view of the operator. ChArUco markers, placed on both the needle and agar jelly phantom, were tracked using RGB cameras built into the ARHMD. A virtual CT slice simulating the needle position was generated on voice command. The application was trialled by senior interventional radiologists and trainee radiologists with a structured questionnaire evaluating face validity and technical aspects.

**Results:**

Sixteen users trialled the application and feedback was received from all. Eleven felt the accuracy and realism was adequate for training and twelve felt more confident about their CT biopsy skills after this training session.

**Discussion:**

The study showed the feasibility of simulating a CT-guided procedure with augmented reality and that this could be used as a training tool.

**Key Points:**

• *Simulating a CT-guided procedure using augmented reality is possible.*

*• The simulator developed could be an effective training tool for clinical practical skills.*

*• Complexity of cases can be tailored to address the training level demands*.

## Introduction

Historically, procedural training in medicine has relied on the application of Halsted’s model of “see one, do one, teach one” [1, 2]. This apprenticeship model of learning is based on observation, then performance, and finally demonstration. This model has some drawbacks: firstly, one cannot reliably and objectively monitor or predict the output of a training program, since feedback is given by the judgement of a trainer. Moreover, it requires that an apprentice learns a procedure by practising on a real patient in a clinical setting, which causes discomfort and may lead to risks for patients, particularly if the procedure involves ionising radiation. It also requires the opportunity to repeat the procedure in a clinical environment until competence is achieved, which makes training accessible only to a select group of students.

Procedural training by simulation can be a risk-free and low-pressure alternative to the apprenticeship method for procedural training [3]. In the former, students can practise a procedure multiple times and can learn from mistakes without risk to patients. The effectiveness of simulations as a training tool for procedural skills has been well-researched over the past few decades [4]. Even low-fidelity simulations in CT procedures have been shown to be effective in reducing procedure time and radiation exposure as well as improving confidence in participants [5, 6]. Now, due to advances in technology, there has been an increased interest in the application of virtual reality (VR) and augmented reality (AR) in procedural training. A key advantage of the use of VR and AR in simulation for procedural training is that these technologies inherently require active learner engagement, which is widely recognized as a cornerstone of effective learning [7]. Recent evidence to support the use of VR and AR simulations in medical education and training is abundant [8–18].

VR and AR simulation for procedural training has also been explored in the domain of radiology. Some examples of this are the use of an AR simulator for ultrasound-guided percutaneous renal access, which showed significant performance improvement in novices [16], and the use of an AR simulator for training in fluoroscopy-guided lumbar puncture, which was regarded as a “realistic replication of the anatomy and procedure” by trial users [13]. These results are promising, but it remains to be shown specifically that CT-guided procedures can be realistically simulated in a scalable and low-cost way.

The Microsoft HoloLens is an augmented reality head-mounted display (ARHMD) with three-dimensional (3D) “mixed reality” capabilities, by which we mean a head-mounted display with an integrated processing unit that allows real-time 3D mapping and tracking of the physical space around the user and that can overlay 3D objects into the field-of-view of the user as part of the physical space. The HoloLens was one of the first ARHMDs with 3D mixed reality capabilities. The second version of the headset, the HoloLens 2, was introduced in 2019. Notably, the HoloLens 2 has a wider field of view and a shifted centre of gravity allowing for improved ergonomics [19]. The device’s visible light camera also has a resolution significantly greater than its predecessor, which can enable more accurate pattern recognition and tracking. We expect that the current capabilities of 3D mixed reality ARHMDs, such as those of the HoloLens 2, together with the haptic feedback from a biopsy phantom, can be used for a realistic simulation of CT-guided procedures, potentially increasing the effectiveness and quality of medical training with a relatively low cost. There are examples of studies examining the feasibility of 3D mixed reality ARHMDs for surgical procedures [20] and for ultrasound-guided interventions [21], but not for the simulation of CT-guided procedures.

To address this, we present a simulator for CT-guided biopsies with haptic feedback using the HoloLens 2 and a bespoke software application. We present the results of a user trial of the simulator in order to evaluate the accuracy and realism, the acceptance by trainers and trainees, and the feasibility of the simulator for the use in the training of CT-guided biopsies.

## Materials and methods

### Phantom

Multiple identical “mock” phantoms of a torso were created from an agar jelly mixture [22]. In each phantom, a reference ChArUco marker was placed in a fixed position for position verification by the ARMHD. A CorVocet© Biopsy Needle was fitted with a ChArUco marker. The needle could be freely manipulated and its movement could be tracked by the ARHMD. The ChArUco markers combine the features of a chessboard pattern, where the exact points of intersection are easily refined, and the ArUco marker family, which facilitates fast detection while allowing for continued detection during partial occlusion of the marker.

### Hardware

The Microsoft HoloLens 2™ is an augmented reality device developed by the Microsoft Corporation. Multiple HoloLens 2 devices were available for use.

### Software

A publicly available CT dataset of a torso was obtained from the Cancer Imaging Archives [19]. A HoloLens application was developed, which displays multiple interactive elements in the field-of-view of the user. The software was written entirely in C# and C++ using Microsoft Visual Studio 2019, the DirectX SDK, and the ChArUco implementation in OpenCV. These elements include an interface containing a rendering of the slices of the CT dataset together with a simulated CT image of a needle, as well as a 3D model of a torso and a 3D model of a needle. The location of the 3D model of the torso corresponds to the reference marker placed on the phantom and the location of the 3D model of the needle corresponds to the marker placed on the biopsy needle. A green line is displayed on the 3D model of the torso to indicate the scan slice location, mimicking the positional laser guide used in modern CT scanners.

The HoloLens application can be controlled by the following voice commands:

1. “Select” selects the needle tool
2. “Scan” brings up the CT scan of the location of the virtual needle
3. “Next” brings up the next CT slice
4. “Previous” brings up the previous CT slice

Image segmentation was used to prepare the CT dataset for the biopsy procedure training. Objects in the rendering of the CT dataset were coloured to indicate the objectives for the participants to target with the biopsy needle. Two retroperitoneal lymph nodes were identified as targets representing two levels of difficulty. The beginner target was a simple pararenal retroperitoneal lymph node with a direct path and the expert target was a para-aortic lymph node.

### Validation

To validate the effectiveness of our simulator for biopsy procedure training, we held a user trial during a day-long introduction to interventional radiology for junior radiology trainees. We enrolled 12 junior trainees and 4 trainers from this event on a voluntary basis. The junior trainees consisted of radiology registrars in training at local NHS medical centres. All participants signed an Imperial College consent form allowing the use of their data for this study.

An introductory presentation was given to all participants, in which the key functionalities of the HoloLens, the relevant voice commands, and relevant gestures for controlling the hardware were introduced.

**Fig. 1:**
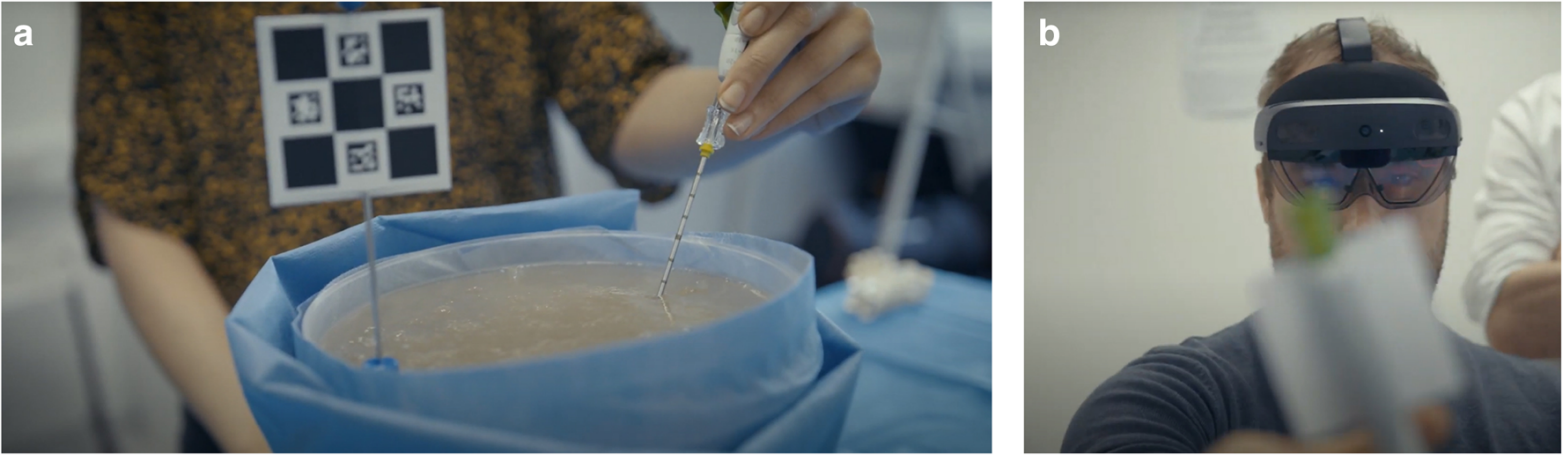
**a** A ChArUco marker in a phantom. **b** A user wearing the HoloLens 2™

**Fig. 2:**
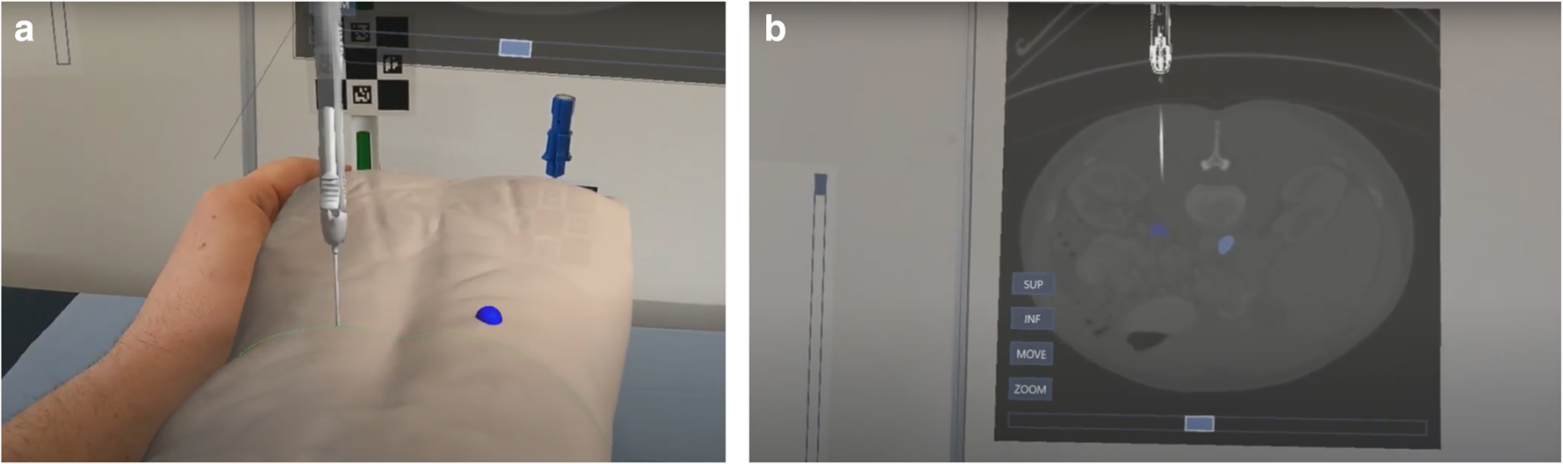
**a** A view of the HoloLens application including a 3D model of a torso and a needle with a ChArUco marker attached. **b** A view of the HoloLens application including a simulated CT image of a needle

Three stations, each consisting of a mock phantom and a HoloLens 2, were available to the participants (Fig. 1). The junior trainees were divided into 4 groups of 3. The participants in the first group were each assigned to a station to complete a session of 30 min, and the groups rotated afterwards. The first 10 min of each session was reserved for an introduction to the HoloLens and the simulator, which included practising voice commands under guidance by an expert. After this introduction, each participant was given 20 min to complete a simulated biopsy procedure, starting with the beginner target and on completion moving to the expert target (Fig. 2).

The procedure that the participants followed consisted of the following steps:

1. Placing the biopsy needle in the mock torso, while aligning the needle with the simulated green (laser) line
2. Taking a simulated scan, advancing the slice position if necessary
3. Viewing the simulated location of the needle in the CT image
4. Adjusting/advancing the needle towards the target
5. Repeating the simulated scan, advancing the slice position if necessary
6. Repeating until the objective is reached

Immediately after the simulation session, participants were asked to complete an anonymous feedback questionnaire evaluating their experience with the augmented reality simulator. The questionnaire consisted of closed-ended questions with Likert-type scale responses and open-ended questions. The questionnaire was constructed out of validated questionnaires from previous studies, but not validated in its current form.

## Results

**Fig. 3:**
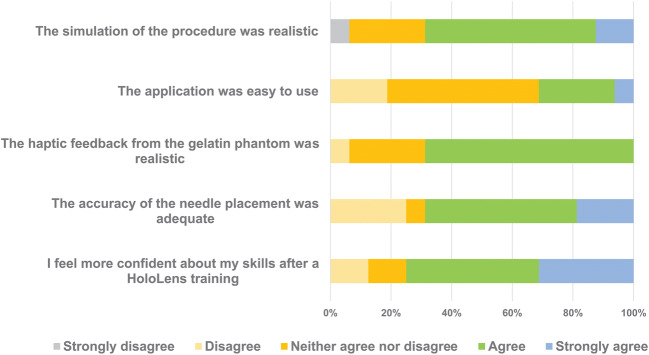
An overview of the responses to a selection of closed-ended questions from the feedback questionnaire

In total, 16 users trialed the application and all of them completed the questionnaire. An overview of the responses to the closed-ended questions is given in Tables 1, 2 and Fig. 3. The results from the feedback questionnaire are presented in terms of accuracy and realism, acceptability, and feasibility for the delivery of biopsy procedure training.

**Table 1:** An overview of the responses to the closed-ended items of the feedback questionnaire

Strongly	Question	n	disagree	Disagree	Neither agree nor disagree	Agree	Strongly agree	Mean (SD)
Acceptability	I enjoyed this training method	16	0 (0%)	0 (0%)	0 (0%)	3 (19%)	13 (81%)	4.8 (0.40)
Acceptability	The application was easy to use	16	0 (0%)	3 (19%)	8 (50%)	4 (25%)	1 (6%)	3.2 (0.83)
Acceptability	I will train and study differently when I know I will be assessed with the HoloLens	16	1 (6%)	2 (13%)	4 (25%)	6 (8%)	3 (19%)	3.5 (1.15)
Acceptability	I feel more confident about my skills after a HoloLens training	16	0 (0%)	2 (13%)	2 (13%)	7 (44%)	5 (31%)	3.9 (1.00)
Acceptability	The HoloLens hindered me to perform the Biopsy procedure	16	5 (31%)	4 (25%)	5 (31%)	2 (13%)	0 (0%)	2.3 (1.06)
Acceptability	This HoloLens app is a good addition to my current training	16	0 (0%)	2 (13%)	1 (6%)	2 (13%)	11 (69%)	4.4 (1.09)
Accuracy	The simulation of the procedure was realistic	16	1 (6%)	0 (0%)	4 (25%)	9 (56%)	2 (13%)	3.7 (0.95)
Accuracy	The accuracy of the needle placement was adequate	16	0 (0%)	4 (25%)	1 (6%)	8 (50%)	3 (19%)	3.6 (1.09)
Accuracy	The haptic feedback from the gelatin phantom was realistic	16	0 (0%)	1 (6%)	4 (25%)	11 (69%)	0 (0%)	3.6 (0.62)
Feasibility	I could demonstrate my skills effectively	16	0 (0%)	4 (25%)	6 (38%)	5 -(31%)	1 -(6%)	3.2 (0.91)
Feasibility	I experienced discomfort wearing the HoloLens	16	11 (69%)	3 (19%)	2-(13%)	0 (0%)	0 (0%)	1.4 (0.73)

**Table 2:** Sub-analysis of expert feedback questionnaire responses on accuracy of simulation

Question	n	Strongly disagree	Disagree	Neither agree nor disagree	Agree	Strongly agree	Mean (SD)
The simulation of the procedure was realistic?	4	0 (0%)	0 (0%)	1 (6%)	2 (13%)	1 (6%)	4.0 (0.82)
The accuracy of the needle placement was adequate?	4	0 (0%)	0 (0%)	1 (6%)	2 (13%)	1 (6%)	4.0 (0.82)
The haptic feedback from the gelatin phantom was realistic?	4	0 (0%)	0 (0%)	0 (0%)	4 (25%)	0 (0%)	4.0 (0.00)

### Accuracy and realism

Accuracy was assessed in terms of needle placement, needle advancement, display of the simulated CT scan of the needle, and “feel” of the mock phantom. Of the users, 11 agreed that the simulation of the procedure was realistic and 11 agreed that the accuracy of the needle placement was adequate. The haptic feedback provided by the agar jelly was perceived as realistic by 11 users. One user stated that the simulation was “as realistic as it can get in a virtual training environment”. In particular, 3 out of 4 experts agreed that the simulation of the procedure was accurate and needle positioning was accurate and all experts agreed that the haptic feedback was realistic (Table 2.)

### Acceptability

More generally, the system should be perceived as an acceptable training tool. All users reported enjoying the training method (13 strongly agreed and 3 agreed). Four users agreed, 8 users neither agreed nor disagreed, and 3 users disagreed with the statement “the application was easy to use”. Fourteen users experienced no discomfort in wearing the HoloLens, and 2 users neither agreed nor disagreed. One user struggled slightly with the hardware and one user commented “I had a few difficulties with the headset in the beginning; figuring out exactly what to say and what it does”.

### Feasibility for biopsy procedure training

Twelve of 16 users agreed that they felt more confident about their skills after training. Furthermore, 13 users agreed with the statement “This HoloLens app is a good addition to my current training”. One user commented that the simulator was beneficial for “understanding the geometrical relationship between needle position and slice number”. One user answered that the simulation provided a “rich new opportunity to practice.”

## Discussion

We have shown that it is possible to simulate a CT-guided procedure using augmented reality technology. In the user trial of the simulator by trainees and trainers, the simulation of the procedure and of the haptic feedback was perceived as realistic by most users (11/16), and most users perceived the accuracy of the needle as adequate (11/16). However, one expert neither agreed nor disagreed that the simulation of the procedure was realistic.

The new functionalities of the HoloLens 2 and its improved ergonomics are likely to have improved scores related to acceptability, as all users reported enjoying the simulation training and no users reported discomfort in wearing the HoloLens. The majority of users neither agreed nor disagreed that the application was easy to use, while the other participants agreed and disagreed on the ease of use in similar proportions.

Most users (12/16) agreed that they felt more confident about their skills after using the simulator and most users (13/16) agreed that the simulator was a good addition to their training. Previous studies of simulations relating to CT-guided procedures also demonstrated an increase in user confidence [5, 6]. Both of these studies also demonstrated a reduced procedure completion time and radiation dose. A limitation of the current study is the fact that procedure time and radiation dose were not calculated.

In the introductory session of 10 min, participants had to learn to use the HoloLens and to interact with the virtual augmented content as well as learn the gestures and voice commands needed to complete the simulation procedure. While this was not measured, we speculate that the inexperience of the participants with both the HoloLens and the procedure itself combined with the short introduction time led to a lower perceived ease-of-use of the simulator, possibly due to an increased cognitive load during the simulation.

Compared to existing simulations, our simulator notably makes use of readily available materials and technologies with bespoke software, resulting in a low cost and high scalability.

Further work is needed to examine the applicability of this technology in training as well as in clinical practice. We suggest examining the effect of the simulator on the performance of biopsy procedures, in particular in comparison to “classical” biopsy procedure training on a real patient.

## Abbreviations and acronyms

3D: Three-dimensional
AR: Augmented reality
ARHMD: Augmented reality head-mounted device
ChArUco: Chessboard Augmented Reality library from the University of Cordoba
CT: Computed tomography
VR: Virtual reality

## Declarations

### Guarantor

The scientific guarantor of this publication is Dimitri Amiras.

### Statistics and biometry

No complex statistical methods were necessary for this paper.

### Methodology

• Prospective

• Experimental study

• Performed at one institution
